# Determinants of clinical trial failure in multiple sclerosis: insights from ClinicalTrials.gov

**DOI:** 10.21203/rs.3.rs-7496530/v1

**Published:** 2025-09-15

**Authors:** Alejandro Rivero-de-Aguilar, Mónica Pérez-Ríos, Joseph S. Ross, Marta Mascareñas-García, Alberto Ruano-Raviña, Leonor Varela-Lema

**Affiliations:** University of Santiago de Compostela; University of Santiago de Compostela; Yale School of Medicine; University of Santiago de Compostela; University of Santiago de Compostela; University of Santiago de Compostela

**Keywords:** multiple sclerosis, clinical trial

## Abstract

**Background.:**

Clinical trial failure —when a study is withdrawn or terminated for reasons unrelated to safety or efficacy—wastes resources and exposes patients to unnecessary risks. We examined such failures in the field of multiple sclerosis (MS).

**Methods.:**

We searched ClinicalTrials.gov for phase III and IV MS drug trials conducted in 2008–2024. Trials were classified as normally ended or failed based on recruitment status and reported reasons. When information was missing, we reviewed publications and contacted principal investigators (PIs). Kaplan-Meier method was used to assess time to trial end and multivariate logistic regression was used to identify factors associated with failure.

**Results.:**

The 282 clinical trials included in our analysis involved 62 experimental drugs, primarily disease-modifying therapies (n = 213 trials). Most studies aimed to evaluate drug efficacy (n = 196 trials). Overall, 74.8% trials ended normally while 25.2% failed. On average, failed trials ended ten months earlier that the rest of studies (trial actual duration: 17.8 vs. 28.2 months, p < 0.001). Main reasons for failure were low recruitment (28.2%), unspecified business decisions (26.8%) and logistical problems (12.7%). In 28.2% of occasions it remained undisclosed. Trials evaluating drug safety (OR 0.35, 95%CI 0.13–0.94) and those with ≥ 50 centres (OR 0.10, 95%CI 0.02–0.38) failed less often. Failure was not associated with other factors such as study start date, PI location or industry involvement.

**Conclusions.:**

One out of four MS clinical trials fails. Increasing the number of study sites and improving recruitment strategies could enhance their success rates.

## INTRODUCTION

Clinical trials are essential for advancing therapeutic developments across all fields of medicine. They serve as the cornerstone of evidence-based practice, providing the rational basis that guides physicians in their clinical decision-making (1). Conducting clinical trials is a time-consuming and expensive process, with a median cost of $21.4 million for phase III studies (2), and with uncertain return on investment, as only 10–20% of the experimental drugs evaluated in humans receiving marketing approval (3) Furthermore, clinical trials rely on the willingness of individuals to voluntarily enrol without a complete certainty of risks and benefits, calling for a strict adherence to the ethical principles of medical research (4).

Studies based on clinical trial registries (5, 6) or ethics committees databases (7) show that 14–27% of finished clinical trials have a premature end, without having collected information for the entirety of the pre-specified patient sample or follow-up period. Trial termination due to safety concerns or lack of efficacy is considered appropriate, since it prevents additional patient exposure to harmful or ineffective therapies and limits further expenditure of resources. However, trial withdrawal or termination because of economic considerations, low recruitment, or logistic or methodological issues is controversial, as it can deprive patients and the community of the benefits of ascertaining information on the clinical issue being studied, despite having already used valuable resources and exposed patients to unnecessary risks. Those trials are said to have failed (8) since they are unlikely to answer the research question despite the best efforts of patients, clinicians, and researchers.

Multiple sclerosis (MS) is a debilitating neurological disease that has been the subject of study for many clinical trials in the last decade, leading to the approval of numerous drugs (9). Clinical research in MS is strongly supported by the pharmaceutical industry but also receives large investments from governments and non-profit organizations (10), making the rational use of assets essential. Some studies have addressed the problem of clinical trial failure to complete in some conditions, such as cancer (11) and cardiovascular diseases (12), but none have focused on MS so far. Understanding the underlying factors could enhance trial design, increase study participation, and save finite resources in MS clinical research.

Therefore, the aim of this study is to explore clinical trial failures in MS studies that have been carried out between 2008 and 2024, assessing its frequency, reasons, and associated factors.

## MATERIAL AND METHODS

### Data source and search strategy

A search was conducted on May 1, 2024 in ClinicalTrials.gov to identify clinical trials carried out between 2008 and 2024. The advanced search engine on the website was used applying the terms “*multiple sclerosis*” and the filters “*phase III*, “*phase IV*”, “*start date on or after 1/1/2008*” and “*primary completion on or before 31/12/2024*”. This search was updated in September, 2024.

### Clinical trial selection

The information pertaining to each trial record was reviewed and its eligibility was established attending to the following criteria. We included, phase III or IV clinical trials examining drugs for MS that had been finished before December 31, 2024. Ongoing studies, trials not limited to MS population, and studies evaluating non-pharmacological interventions or diagnostic procedures were excluded. A trial was considered to be finished if its recruitment status at ClinicalTrials.gov was listed “*completed*” (i.e. the study ended as planned), “*terminated*” (i.e. the study ended prematurely), “*withdrawn*” (i.e. the study ended before any participant was ever enrolled) or “*unknown*” (i.e. the study was said to be ongoing but has passed its estimated completion date for two or more years without an update in its status). Trials were considered to be “*ongoing*” if they were listed “*recruiting*”, “*enrolling by invitation*”, “*active, not recruiting*” or “*suspended*” (i.e. temporarily stopped). Definitions for these recruitment statuses can be found in ClinicalTrials.gov’s glossary of terms (13). Recruitment status for classified as “*unknown*” and “*ongoing*” was double-checked, since ClinicalTrials.gov is occasionally outdated (14). Efforts were also made to investigate the reasons for trial termination or withdrawal when these were not explicitly reported in the registry. To this end, consecutive searches in PubMed, EMBASE, and Google Scholar were conducted using the study title, trial registration number, and drug name to locate publications reporting on the trials’ outcomes. Links to publications posted at ClinicalTrials.gov were also reviewed. If no relevant information was found, the principal investigators were contacted via e-mail up to three times.

### Data extraction

Data available at ClinicalTrials.gov were extracted using a predesigned Microsoft Excel worksheet. Extracted information comprised drug name, recruitment status (including reasons for termination or withdrawal), study phase, study aim, number of arms, assignment, randomization, comparator group, masking, eligibility criteria (sex, age, maximum allowed disability, and MS phenotype being studied), primary outcome measure definition, estimated sample size, participating centres, sponsors and collaborators, principal investigator location and study dates (“*study registration first meeting quality control criteria*”, “*estimated study start*”, “*actual study start*”, “*estimated primary completion*”, and “*actual primary completion*”). Definitions can be found in ClinicalTrials.gov’s glossary of terms (13).

Experimental drugs were classified into four categories according to their mechanism of action: “*disease modifying therapy*” (DMT) (targeting the underlying disease pathophysiology to prevent future relapses and/or accrual of disability), “*relapse treatment*” (accelerating the functional recovery from a clinical relapse), “*symptomatic drug*” (managing specific MS symptoms, such as spasticity, fatigue or pain), and “*other*” (unclassifiable under the previous categories; for example, drugs reducing the systemic or local side effects of DMTs).

Disability was classified into three categories, according to the baseline “*Expanded Disability Status Scale*” (EDSS) score (15): mild (EDSS < 4.0; no walking impairment), moderate (EDSS 4.0–5.5), and severe (EDSS > 5.5; requires a walking aid to walk about 100 m).

Four categories of clinical trials were defined according to the MS phenotype included in the studies: those evaluating relapsing forms (i.e. clinically isolated syndrome (CIS), relapsing-remitting MS, and clinically active secondary progressive MS); those evaluating non-relapsing forms (i.e. clinically inactive secondary progressive MS and primary progressive MS); those accepting patients with both relapsing and non-relapsing forms; and those restricted to patients with radiologically isolated syndrome (RIS). This classification is inspired by the disease phenotypes defined by Lublin in 2014 (16) and aims to distinguish patients with more inflammatory forms of MS from those in whom disability accrual occurs primarily due to neurodegeneration. CIS was included within relapsing forms since, following the inclusion of CSF oligoclonal bands in the 2017 McDonald diagnostic criteria (17), many patients who were previously classified as CIS would nowadays be considered as having relapsing-remitting MS.

### Data analysis

A descriptive analysis was conducted. Qualitative variables were expressed as absolute frequency (n) and relative frequency (%). Quantitative variables were expressed as median and interquartile range (IQR). Completed clinical trials and studies that were alleged to be terminated or withdrawn due to safety, efficacy, or other legitimate reasons were classified as “*normally ended*”. Trials terminated or withdrawn due to low recruitment, high number of dropouts, inadequate trial design, logistical problems, ethical concerns, unspecified business decisions, or unreported reasons, and those with an unknown recruitment status were classified as “*failed*”. Trials were classified independently by two authors and disagreements were resolved by consensus. Estimated trial duration was calculated as the interval between the originally estimated study start date and the originally estimated primary completion. When the principal investigator’s geographical location was not available but the trial was restricted to a particular area (e.g. Europe), he/she was assumed to be located within that area. Trial registration status was classified as prospective or retrospective if it was done before or after the inclusion of the first participant, respectively.

To conduct comparisons between groups, Fisheŕs exact test or χ^2^ test were applied to nominal variables and Mann-Whitneýs *U* test to ordinal variables. A two-tailed p-value of less than 0.05 was considered statistically significant. When significant differences between normally ended and failed trials were found, simple and multivariate logistic regression were used to calculate crude and adjusted odds ratios (OR), along with their 95% confidence intervals (95% CI). Kaplan-Meier method and log rank test were used to assess time to trial end. Time to trial end was defined as the number of months from the studýs actual start date until its actual primary completion date or until the study status changed to “*withdrawn*”. When the actual start date was not available, the most recent estimated start date was used. When the actual primary completion date was not available, the most recent estimated primary completion date was used. Data was analysed with SPSS 27.0.1 for Mac (SPSS Inc, Chicago, IL).

## RESULTS

### Search process

**Figure 1:**
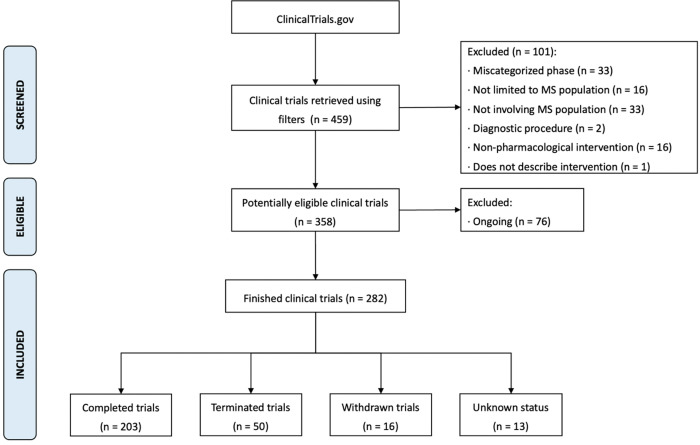
Flowchart depicting the step-by-step process to select the studies included in the analysis. MS: multiple sclerosis

Through the advanced search in ClinicalTrials.gov, 459 clinical trials were retrieved. One hundred and one studies were excluded after reading the study description in the registry and verifying they did not meet the eligibility criteria (figure 1). Seventy-six more were excluded because they were still ongoing. Thus, 282 clinical trials were included in the analysis (**supplementary material**).

### Clinical trials’ characteristics

**Figure 2:**
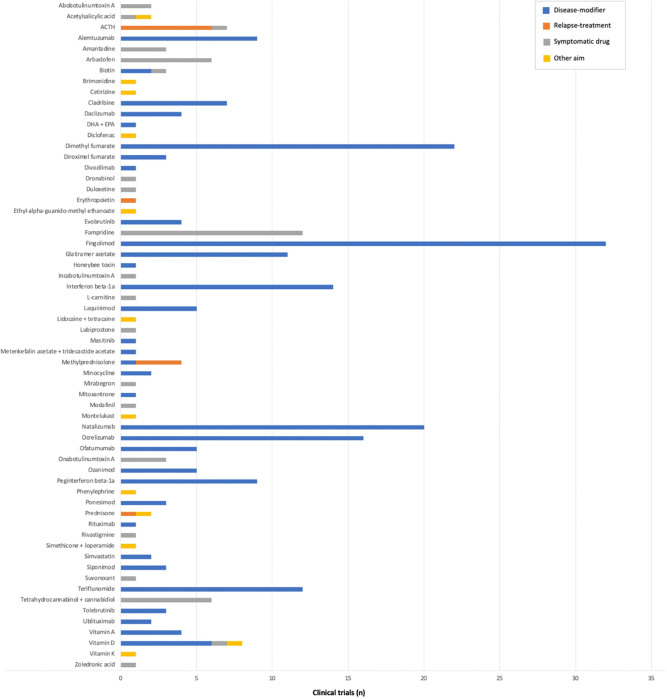
Experimental drugs for multiple sclerosis evaluated in the clinical trials. ACTH: adrenocorticotropic hormone. DHA: docosahexaenoic acid. EPA: eicosahexanoic acid.

Overall, the clinical trials included in our analysis evaluated 62 drugs, with fingolimod being the most common (n = 32), followed by dimethyl fumarate (n = 22) and natalizumab (n = 20) (figure 2). Around half of them were phase III (n = 145, 51.4%) and half were phase IV (n = 137, 48.6%) (table 1). The majority of these trials (n = 197, 69.9%) were aimed at evaluating the drug efficacy. Most studies were controlled (n = 187, 66.1%) and, among these, most of them were randomized (n = 163, 87.2%) but only one-third used an active comparator (n = 59, 31.6%). Nearly all trials were limited to adult population (n = 270, 95.7%), one-third was restricted to patients with mild to moderate disability (n = 100, 35.4%) and two-thirds were restricted to patients with relapsing forms of MS (n = 193, 68.4%). Most studies were multicentre (n = 208, 73.8%) and nearly half (n = 114, 42.7%) were international. Two-thirds of the studies had a clinical primary outcome measure (e.g. annualized relapse rate or incidence of treatment-emergent adverse events) (n = 179, 63.5%), and the remaining used a paraclinical outcome (n = 74, 26.2%) (e.g. number of new/newly enlarged T2 MRI lesions; number of participants who developed neutralizing antibodies; or absolute change in global retinal nerve fibre layer thickness) or a combination of clinical and paraclinical outcomes (n = 29, 10.3%) (e.g. absolute change from baseline EDSS and number of active lesions in MRI as co-primary outcomes; or proportion of patients converting to multiple sclerosis according to McDonald 2010 criteria). One-tenth of the trials used a composite primary outcome (n = 31, 11.0%) (e.g. proportion of patients losing the *“No Evidence of Disease Activity 3”* status; or time to first event of clinical and/or radiological disease activity). The median estimated sample size was 193 individuals (IQR 60 – 518) and the median estimated trial duration was 24.6 months (IQR 14.5 – 38.3). Pharmaceutical industry involvement, either sponsorship or collaboration, was common (n = 229, 81.2%). Registration in ClinicalTrials.gov was prospective in more than half of the trials (n = 165, 58.5%).

### Methodological differences between phase III and phase IV clinical trials

A comparative analysis of the methodological characteristics of phase III and phase IV clinical trials is presented in **supplementary table 1**. Statistically significant differences were observed in terms of drug type (p = 0.003) and study aim (p < 0.001), primarily due to a higher proportion of phase III trials evaluating symptomatic drugs (20.0% vs. 12.4%), and a higher proportion of phase IV trials examining aspects other than safety and efficacy (20.4% vs. 2.8%). Compared to phase III clinical trials, phase IV trials were less frequently randomised (42.3% vs. 72.4%, p < 0.001), less frequently controlled with an active agent (16.1% vs. 25.5%, p = 0.002), less frequently multicentre (55.9% vs. 86.8%, p < 0.001), and less frequently international (18.0% vs. 65.5%, p < 0.001). Conversely, phase IV trials more frequently had estimated sample sizes below 100 participants (53.3% vs. 13.8%, p < 0.001), more frequently had an estimated duration shorter than two years (62.1% vs. 31.1%, p < 0.001), and more frequently used paraclinical primary outcomes (37.2% vs. 15.9%, p < 0.001). Regarding sponsorship and collaboration, industry was more often involved in phase III trials (87.6% vs. 74.5%, p = 0.005), whereas universities and hospitals were more commonly involved in phase IV trials (19.7% vs. 7.6%, p = 0.003; 30.7% vs. 11.7%, p < 0.001).

### Reasons and timing of clinical trial end

According to our criteria, 211 trials (74.8%) were classified as normally ended whilst 71 (25.2%) were considered to have failed. The most common reason for study end within the first group was the trial completion (n = 203, 96.2%) (table 2). Regarding failed trials, low recruitment (n = 20, 28.2%), unspecified business decision (n = 19, 26.8%) and logistical problems (n = 9, 12.7%) were the most common reported causes of trial end, but nearly one third of these failed studies (n = 20, 28.2%) did not specify any reason.

Overall, no significant differences were observed in the distribution of reasons for trial failure when studies were dichotomised by trial phase (**supplementary table 2**), experimental drug (**supplementary table 3**), eligibility criteria (**supplementary table 4**), or primary outcome (**supplementary table 5**). The sole exceptions were a lower proportion of failures attributed to inadequate trial design in phase IV trials compared to phase III studies (0.0% vs. 16.1%, p = 0.013) and in trials accepting patients with severe disability compared to those that did not (0.0% vs. 21.7%, p = 0.004); significant differences in the proportion of failures attributable to logistical problems depending on drug type (DMT: 21.7% vs. relapse treatment: 60.0% vs. symptomatic drug: 0.0% vs. other: 20.0%, p = 0.019); and significant differences in the proportion of failures attributed to low recruitment according to the primary outcome (clinical: 35.0% vs. paraclinical: 12.0% vs. both: 50.0%, p = 0.042).

**Figure 3:**
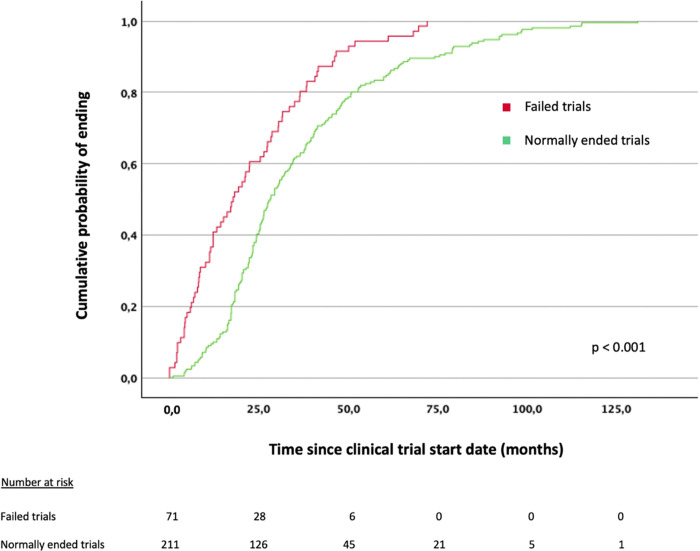
Kaplan-Meier plot assessing time to study end in normally ended vs. failed trials.

**Figure 4:**
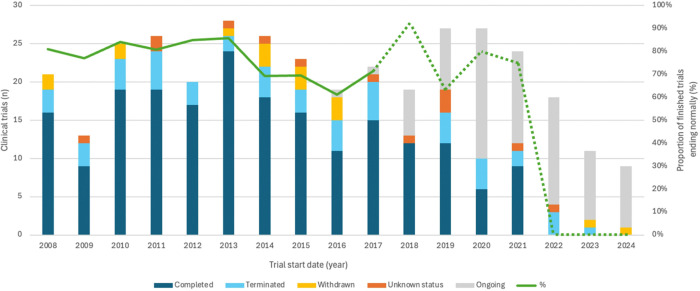
Evolution of recruitment status and clinical trial failure through time (cut-off date: September, 2024). When the actual trial start date was not available, the most recent estimated start date was used. The green line represents the proportion of finished trials that have ended normally (note it has been dotted from year 2018 onwards because of the large number of ongoing studies). Trials started in recent years could be underrepresented because of pending study registration. Definitions are provided in the body of the article.

The median time from the trial start to its end was shorter for failed trials than for those ending normally (17.8 vs. 28.2 months; p < 0.001) (figure 3). Evolution of recruitment status and clinical trial failure through time is represented in figure 4, in which there seems to be a trend towards a lower proportion of trials ending normally over time.

### Factors associated with clinical trial failure

As shown in table 1, statistically significant differences between normally ended and failed trials were found in terms of five variables: study aim (p = 0.037), number of participating centres (p < 0.001), international nature of the study (p = 0.032), trial start date (p = 0.025), and type of registration in ClinicalTrials.gov (p = 0.008).

Bivariate analysis (table 3) showed that trials less likely to fail were those aimed to study the safety of the experimental drug (OR 0.36, 95% CI 0.15 – 0.83), those with 50 or more participating centres (OR 0.18, 95% CI 0.07 – 0.49), and international studies (OR 0.51, 95% CI 0.28 – 0.95). On the other hand, trials more likely to fail were those started in 2016 onwards (OR 1.87, 95% CI 1.08 – 3.23) and those prospectively registered in ClinicalTrials.gov (OR 2.17, 95% CI 1.21 – 3.89). After conducting a multivariate analysis that included those five variables (table 3), two of them kept their statistical significance: studies aimed to assess the safety of the experimental drug (OR 0.35, 95% CI 0.13 – 0.94) and trials with 50 or more participating centres (OR 0.10, 95% CI 0.02 – 0.38). We performed a post-hoc analysis to assess if the association between the number of participating centres and clinical trial failure was influenced by the international nature of the study, and we found no interaction (p = 0.996). Neither of the adjusted ORs changed significantly when the variables “*study phase*” and “*industry involvement*” were included in a second multivariate analysis (**supplementary table 6**).

Although we did not observe a statistically significant difference in the overall percentage of failed trials between phase III and phase IV studies (21.4% vs. 29.2%, p = 0.131), we decided to conduct a post-hoc analysis by subcategorising each trial phase. No significant differences were observed between phase III trials assessing efficacy (which typically seek regulatory approval to market the drug) and the remaining phase III trials (24.1% vs. 13.5%, p = 0.176). However, we did observe a statistically significant difference between phase IV trials that were extension studies and the remaining phase IV trials (0.0% vs. 31.5%, p = 0.034). This difference within phase IV trials was no longer statistically significant after performing a multivariate analysis (**supplementary table 7**).

## DISCUSSION

To our knowledge, this is the first study comprehensively assessing clinical trial failure in the field of MS. After conducting a search on ClinicalTrials.gov, we identified 282 phase III and IV trials that evaluated drugs for MS and were conducted between 2008 and 2024. We found out that a quarter of them could be considered to have failed, since they were not completed as planned, nor were withdrawn or terminated due to safety or efficacy reasons.

ClinicalTrials.gov has been previously used to appraise trial completion rate in cardiovascular medicine (12, 18–20), oncology (11, 21, 22), traumatology (23, 24), surgery (25), and paediatrics (26). In these fields, the proportion of trials failing to be completed ranged from 7.7% (23) to 30.4% (18). However, relevant methodological differences among these studies should be borne in mind: some estimations were not limited to phase III and IV trials (12, 18–24, 26), others included ongoing trials (11, 20), and others excluded trials classified as “*withdrawn*” (20–24, 26) or “*unknown*” in the registry (11, 12, 18–26). Within the few studies that specifically appraised reasons of trial end (11, 21, 22), the proportion of failure as per our definition ranged from 13.9% (21) to 23.0% (22) among finished trials, which is slightly lower than our findings for MS. Thus, there is a need to reflect on why MS clinical trials fail and what can be done to prevent it.

In our study, the most commonly reported reason for trial failure was low recruitment. This finding aligns with what has been previously published in other fields of medicine (5, 6, 23–26, 7, 11, 12, 18–22). Difficulties in recruitment can be caused by several reasons, such as low disease prevalence, narrow selection criteria, mistrust of experimental drugs, high burden of work for patients and recruiters, competition with other trials, and improvements in the standard of therapy (27, 28). When recruitment is low, the trial might become underpowered and be prematurely ended because of futility. To counteract this, the sponsor may expand the number of study locations and allocate additional funds to the research (29). Interestingly, a systematic review aimed to identify strategies to improve recruitment of clinical trials (30) found two that were considered to be effective with a high-certainty of evidence: following an open label design (compared to patient-blinded trials) and using telephone reminders to people who do not respond to postal invitations (compared to not using any reminder). The second most common reason for trial failure in MS was an unspecified business decision, which is consistent with the fact that 81.2% of the trials analysed in our study were sponsored or conducted in collaboration with the pharmaceutical industry. Shifts in business priorities may be influenced by competitive developments or changes in market forecasts, but can also be due to unfavourable interim results concealed under this euphemism. Therefore, these results should be interpreted with caution, and greater transparency in the industry’s decision-making process to discontinue trials should be encouraged.

Our multivariate analysis revealed that trials with certain characteristics were less likely to fail. Studies aimed at assessing drug safety had a 64% lower risk of failure compared to those evaluating drug efficacy. This is probably because of two reasons. First, patient recruitment tends to be less problematic in safety studies as their eligibility criteria are less restrictive. Additionally, patients are often more willing to participate once the drug’s efficacy has already been demonstrated. Second, safety studies are usually less complex from a methodological perspective (e.g. safety outcomes, such as the incidence or severity of adverse events, are typically more straightforward to define and measure than efficacy outcomes). In addition, once the experimental treatment has demonstrated efficacy, it is more likely that the pharmaceutical industry allocates more resources to that promising line of research. However, we did not observe differences in the overall percentage of trial failures between phase III and IV clinical trials. We speculate this may be because most MS clinical trials included in our analysis had industry sponsorship or collaboration (81.2%), which could have minimized the variability in design characteristics typically associated with study phase. The only distinguishing feature was a lower proportion of failures attributed to inadequate trial design in phase IV studies, probably due to their greater methodological simplicity (for example, many were single-group studies).

Similarly, trials involving 50 or more participating centres were associated with a 90% lower likelihood of failure compared to single-centre studies. Multicentre trials generally benefit from a larger pool of potential participants and are less affected by local recruitment issues. A broader network of recruiting centres also allows for greater flexibility, helping mitigate delays and researcher dropouts. The protective effect of a larger number of participating centres is consistent with prior studies in other fields (5, 11, 21, 22). In our analysis, it remained significant even after adjusting for the international nature of trials, underscoring the robustness of the association. Therefore, expanding the number of participating centres in MS trials could be a good strategy to enhance study feasibility and completion rates.

Adaptive trial designs might be another promising tool to mitigate trial failure. These designs allow for pre-specified modifications to the trial protocol based on interim data analyses (31), such as reallocating resources to the most promising study arms or optimizing selection criteria in real-time. Furthermore, adaptive trials align well with ethical principles by minimizing participant exposure to ineffective or harmful treatments. However, they require meticulous planning to prevent biases and warrant special statistical methods to produce valid results.

Two main limitations should be noted when interpreting the findings of our study. First, we only analysed trials registered in ClinicalTrials.gov. However, we consider it provided a representative sample since it is the largest existing clinical trial registry, with more than 533,000 registered studies from 229 countries and territories as of April 2025 (32). Second, reasons for trial withdrawal or termination could not always be retrieved. Nevertheless, we tried to minimize the missing data in the registry by conducting a comprehensive bibliographic search and e-mailing principal investigators.

## CONCLUSIONS

This study shows that MS clinical trials are prone to fail, with one out of four trials being withdrawn or terminated due to reasons unrelated to safety or efficacy. Trials assessing drug safety and those involving 50 or more centres were less likely to fail compared to trials evaluating efficacy and single-centre studies. Increasing the number of study locations could prevent low recruitment, which is the main reported reason for trial failure in our analysis. In any case, efforts should be made to increase the success rate of MS clinical trials, thereby maximising the use of limited resources for the benefit of patients and the medical community.

## Supplementary Material

Supplementary Files

This is a list of supplementary files associated with this preprint. Click to download.

• Supplementarymaterial.docx

## Figures and Tables

**Table 1.** Description of the clinical trials.

Characteristics	All trials (n = 282)	Normally ended (n = 211)	Failed (n = 71)	p-value

**Drug type**, n (%)				NS
Disease modifying therapy	213 (75.5)	160 (75.8)	53 (74.6)	
Relapse treatment	11 (3.9)	6 (2.8)	5 (7.0)	
Symptomatic drug	46 (16.3)	38 (18.0)	8 (11.3)	
Other	12 (4.3)	7 (3.3)	5 (7.0)	

**Add-on therapy**, n (%)				NS
Yes	27 (9.6)	18 (8.5)	9 (12.7)	
No	255 (90.4)	193 (91.5)	62 (87.3)	

**Study phase**, n (%)				NS
III	145 (51.4)	114 (54.0)	31 (43.7)	
IV	137 (48.6)	97 (46.0)	40 (56.3)	

**Study aim**, n (%)				0.044
Efficacy	197 (69.9)	138 (65.4)	59 (83.1)	
Safety	53 (18.8)	46 (21.8)	7 (9.9)	
Satisfaction	9 (3.2)	8 (3.8)	1 (1.4)	
Other	23 (8.2)	19 (9.0)	4 (5.6)	

**Study arms**, n (%)				NS
1	95 (33.7)	72 (34.1)	23 (32.4)	
2	149 (52.8)	113 (53.6)	36 (50.7)	
3	30 (10.6)	21 (10.0)	9 (12.7)	
≥ 4	8 (2.8)	5 (2.4)	3 (4.2)	

**Assignment**, n (%)				NS
Single group	95 (33.7)	72 (34.1)	23 (32.4)	
Parallel	170 (60.3)	125 (59.2)	45 (63.4)	
Crossover	17 (6.0)	14 (6.6)	3 (4.2)	

**Randomization**, n (%)				NS
Yes	163 (57.8)	121 (57.3)	42 (59.2)	
No	119 (42.2)	90 (42.7)	29 (40.8)	

**Comparator group**, n (%)				NS
Active agent	59 (20.9)	43 (20.4)	16 (22.5)	
Other than active agent	128 (45.4)	96 (45.5)	32 (45.1)	
None	95 (33.7)	72 (34.1)	23 (32.4)	

**Masking**, n (%)				NS
Open label	143 (50.7)	107 (50.7)	36 (50.7)	
Single blind	11 (3.9)	9 (4.3)	2 (2.8)	
Double blind	34 (12.1)	21 (10.0)	13 (18.3)	
Triple blind	32 (11.3)	23 (10.9)	9 (12.7)	
Quadruple blind	62 (22.0)	51 (24.2)	11 (15.5)	

**Age group**, n (%)				NS
< 18 years	6 (2.1)	4 (1.9)	2 (2.8)	
≥ 18 years	270 (95.7)	203 (96.2)	67 (94.4)	
Both	6 (2.1)	4 (1.9)	2 (2.8)	

**Sex**, n (%)				NS
Males	1 (0.4)	0 (0.0)	1 (1.4)	
Females	1 (0.4)	1 (0.5)	0 (0.0)	
Both	280 (99.3)	210 (99.5)	70 (98.6)	

**Maximum allowed disability**, n (%)				NS
Mild (EDSS <4.0)	4 (1.4)	4 (1.9)	0 (0.0)	
Moderate (EDSS 4.0 – 5.5)	96 (34.0)	73 (34.6)	23 (32.4)	
Severe (EDSS >5.5)	182 (64.5)	134 (63.5)	48 (67.6)	

**Disease phenotype**, n (%)				NS
Relapsing forms ^1^	193 (68.4)	140 (66.4)	53 (74.6)	
Non-relapsing forms ^2^	9 (3.2)	8 (3.8)	1 (1.4)	
Relapsing and non-relapsing forms	77 (27.3)	61 (28.9)	16 (22.5)	
Radiologically isolated syndrome	3 (1.1)	2 (0.9)	1 (1.4)	

**Number of participating centres**, n (%)				< 0.001
1	74 (28.1)	51 (24.6)	23 (41.1)	
2 – 9	39 (14.8)	27 (13.0)	12 (21.4)	
10 – 49	72 (27.4)	57 (27.5)	15 (26.8)	
≥ 50	78 (29.7)	72 (34.8)	6 (10.7)	

**International study**, n (%)				0.032
Yes	114 (42.7)	96 (46.2)	18 (30.5)	
No	153 (57.3)	112 (53.8)	41 (69.5)	

**Originally estimated sample size**, n (%)				NS
< 100	93 (33.0)	63 (29.9)	30 (42.3)	
≥ 100	189 (67.0)	148 (70.1)	41 (57.7)	

**Number of primary outcomes**, n (%)				NS
One	206 (73.0)	149 (70.6)	57 (80.3)	
Several	76 (27.0)	62 (29.4)	14 (19.7)	

**Type of primary outcome** ^3^, n (%)				NS
Clinical	179 (63.5)	139 (65.9)	40 (56.3)	
Paraclinical	74 (26.2)	49 (23.2)	25 (35.2)	
Both	29 (10.3)	23 (10.9)	6 (8.5)	

**Patient-reported primary outcome**, n (%)				NS
Yes	50 (17.7)	42 (19.9)	8 (11.3)	
No	282 (82.3)	169 (80.1)	63 (88.7)	

**Composite primary outcome**, n (%)				NS
Yes	31 (11.0)	22 (10.4)	9 (12.7)	
No	251 (89.0)	189 (89.6)	62 (87.3)	

**Trial start date**, n (%)				0.025
2008 – 2015	182 (64.5)	144 (68.2)	38 (53.5)	
2016 – 2024	100 (35.5)	67 (31.8)	33 (46.5)	

**Estimated trial duration**, n (%)				NS
< 2 years	118 (46.1)	86 (45.3)	32 (48.5)	
≥ 2 years	138 (53.9)	104 (54.7)	34 (51.5)	

**Sponsors and collaborators** ^3^, n (%)				
University	38 (13.5)	25 (11.8)	13 (18.3)	NS
Hospital	59 (20.9)	39 (18.5)	20 (28.2)	NS
Industry	229 (81.2)	171 (81.0)	58 (81.7)	NS
Government / public institution	5 (1.8)	5 (2.4)	0 (0.0)	NS
Other	7 (2.5)	4 (1.9)	3 (4.2)	NS

**Principal investigator location**, n (%)				NS
USA or Canada	82 (41.2)	57 (38.5)	25 (49.0)	
Europe ^4^	86 (43.2)	69 (46.6)	17 (33.3)	
Other	31 (15.6)	22 (14.9)	9 (17.6)	

**ClinicalTrials.gov registration**, n (%)				0.008
Prospective	165 (58.5)	114 (54.0)	51 (71.8)	
Retrospective	117 (41.5)	97 (46.0)	20 (28.2)	

EDSS: Expanded Disability Status Scale. NS: not significant.

1 Clinically isolated syndrome (CIS) and/or relapsing-remitting multiple sclerosis (RR-MS) and/or clinically active secondary progressive multiple sclerosis (SP-MS).

2 Clinically inactive secondary progressive multiple sclerosis (SP-MS) and/or primary progressive multiple sclerosis (PP-MS).

3 Several options can apply for the same trial.

4 Schengen area countries, UK and Ireland.

**Table 2.** **Reasons for trial ending** (note that several reasons can apply to the same trial).

**Normally ended trials** (n = 211), n (%)	
Trial completion	203 (96.2)
Efficacy reasons	5 (2.4)
Safety reasons	0 (0.0)
Other *	3 (1.4)

**Failed trials** (n = 71), n (%)	
Low recruitment	20 (28.2)
High number of dropouts	0 (0.0)
Inadequate trial design	5 (7.0)
Logistical problems	9 (12.7)
Ethical concerns	1 (1.4)
Unspecified business decision	19 (26.8)
Other **	1 (1.4)
Reason not provided	20 (28.2)

* No longer needed based on recent publications (n = 1), sufficient data were collected (n = 2)

** Participants recruited to a different study (n = 1)

**Table 3.** Factors associated with clinical trial failure

Characteristics	Bivariate analysis			Multivariate analysis*		
	
	OR	95% CI	p-value	OR	95% CI	p-value

**Study aim**						
Efficacy	1	-	-	1	-	-
Safety	0.36	0.15 – 0.83	0.017	0.35	0.13 – 0.94	0.036
Satisfaction	0.29	0.04 – 2.39	NS	0.66	0.07 – 6.31	NS
Other	0.49	0.16 – 1.51	NS	0.55	0.17 – 1.82	NS

**Number of participating centres**						
1	1	-	-	1	-	-
2 – 9	0.99	0.43 – 2.28	NS	0.87	0.36 – 2.12	NS
10 – 49	0.58	0.27 – 1.24	NS	0.43	0.15 – 1.07	NS
≥ 50	0.18	0.07 – 0.49	< 0.001	0.10	0.02 – 0.38	< 0.001

**International study**						
No	1	-	-	1	-	-
Yes	0.51	0.28 – 0.95	0.034	1.62	0.58 – 4.47	NS

**Trial start date** (year)						
2008 – 2015	1	-	-	1	-	-
2016 – 2024	1.87	1.08 – 3.23	0.026	1.83	0.95 – 3.53	NS

**ClinicalTrials.gov registration**						
Retrospective	1	-	-	1	-	-
Prospective	2.17	1.21 – 3.89	0.009	2.01	0.98 – 4.12	NS

* Adjusted by the variables *“study aim”, “number of participating centres”, “international study”, “trial start date”* and “ClinicalTrial.gov *registration*”.

95% CI: 95% confidence interval. NS: not significant. OR: odds ratio.

## Data Availability

The full list of clinical trials included in the analysis is available as supplementary material.

## Abbreviations

95%CI: 95% confidence interval
CIS: Clinically Isolated Syndrome
CSF: cerebrospinal fluid
DMT: Disease Modifying Therapy
EDSS: Expanded Disability Status Scale
IQR: interquartile range
MS: multiple sclerosis
n.: number
NS: not significant
OR: odds ratio
RIS: Radiologically Isolated Syndrome
MRI: magnetic resonance imaging
